# LlR3MYB-mediated flavonoid biosynthesis confers cold stress tolerance in *Lilium lancifolium* through the LlDREB-LlCHS2 regulatory cascade

**DOI:** 10.1093/hr/uhag065

**Published:** 2026-02-27

**Authors:** Yubing Yong, Heng Bi, Mingyue Li, Yichao Zhu, Qi Zhou, Wen Xing, Sixiang Zheng, Lin Zhang, Yingmin Lyu, Rong Song

**Affiliations:** Hunan Big Data Engineering Technology Research Center of Natural Protected Areas Landscape Resources, Yuelu Mountain Laboratory of Hunan Province, Central South University of Forestry and Technology, Changsha, Hunan 410004, China; Hunan Big Data Engineering Technology Research Center of Natural Protected Areas Landscape Resources, Yuelu Mountain Laboratory of Hunan Province, Central South University of Forestry and Technology, Changsha, Hunan 410004, China; Hunan Big Data Engineering Technology Research Center of Natural Protected Areas Landscape Resources, Yuelu Mountain Laboratory of Hunan Province, Central South University of Forestry and Technology, Changsha, Hunan 410004, China; Hunan Big Data Engineering Technology Research Center of Natural Protected Areas Landscape Resources, Yuelu Mountain Laboratory of Hunan Province, Central South University of Forestry and Technology, Changsha, Hunan 410004, China; Hunan Big Data Engineering Technology Research Center of Natural Protected Areas Landscape Resources, Yuelu Mountain Laboratory of Hunan Province, Central South University of Forestry and Technology, Changsha, Hunan 410004, China; Hunan Big Data Engineering Technology Research Center of Natural Protected Areas Landscape Resources, Yuelu Mountain Laboratory of Hunan Province, Central South University of Forestry and Technology, Changsha, Hunan 410004, China; Hunan Institute of Nuclear Agriculture and Chinese Medicinal Materials, Hunan Academy of Agricultural Sciences, Changsha, Hunan 410125, China; Key Laboratory of Cultivation and Protection for Non-wood Forest Trees, Ministry of Education, Central South University of Forestry and Technology, Changsha, Hunan 410004, China; Beijing Key Laboratory of Ornamental Germplasm Innovation and Molecular Breeding, National Engineering Research Center for Floriculture, College of Landscape Architecture, Beijing Forestry University, Beijing 100083, China; Hunan Institute of Nuclear Agriculture and Chinese Medicinal Materials, Hunan Academy of Agricultural Sciences, Changsha, Hunan 410125, China

## Abstract

Lilies (*Lilium* spp.) are globally important ornamental crops which are constrained by their narrow thermal tolerance range. However, tiger lily (*Lilium lancifolium*), a wild lily species, exhibits remarkable cold tolerance. Based on our previous findings, we proposed that LlR3MYB, an R3-MYB transcription factor (TF), confers cold tolerance via transcriptional regulation of flavonoid metabolism in tiger lily. Here, we revealed that LlR3MYB represents a unique CPC-type R3-MYB TF exhibiting a bifunctional role in flavonoid metabolism. Specifically, LlR3MYB suppresses anthocyanin biosynthesis while promoting non-anthocyanin flavonoid accumulation (i.e. flavonols, flavones, and chalcones) responding to cold stress. Overexpression of *LlR3MYB* in tobacco and tiger lily increased total flavonoid content but reduced anthocyanin levels, consistent with the upregulation of early biosynthesis genes (e.g. *CHS* and *FLS*) and repression of late biosynthesis genes (e.g. *DFR* and *ANS*) in the pathway. In contrast, silencing *LlR3MYB* in tiger lily reduced total flavonoid production, enhanced anthocyanin accumulation, and compromised cold resistance. Mechanistically, LlR3MYB can directly bind to the AC-I element (ACCTACC) and MBSI motif (CAACGGTT) in the *LlCHS2* promoter and activating its transcription, with enhanced activation under low temperature conditions. Mutations of critical residues within the C1/C2 repressor motifs may endow LlR3MYB with this transcriptional activation function. Furthermore, LlDREB can directly bind to the DRE motif (ACCGAC) in the *LlR3MYB* promoter and activating its transcription in a low-temperature-dependent manner. Our findings uncover a branch-specific regulatory mechanism by which MYB TFs fine-tune flavonoid biosynthesis, highlighting their essential role in plant cold stress responses.

## Introduction

Low temperature represents a significant abiotic stressor that negatively impacts plant growth and development, restricts geographic distribution, and causes substantial yield reductions in agricultural and horticultural crops [1]. Cold stress can interfere with the initial plant growth by altering the water status of cells, due to ion imbalance and hypertonicity [2]. Subsequent to ionic toxicity, the burst of reactive oxygen species (ROS) results in decreased photosynthetic activity, nucleic acid damage, lipid peroxidation, protein oxidation, and enzyme inhibition, ultimately leads to programmed cell death [3, 4]. To avert the oxidative stress accompanied by cold stress, plants have evolved a dual-layered antioxidant defense system involving both antioxidative enzymes and non-enzymatic antioxidants [1, 5]. Among them, flavonoids serve as crucial antioxidants by directly scavenging ROS, upregulating antioxidant enzyme activity, and inhibiting oxidases [6, 7].

Flavonoids are water-soluble polyphenolic compounds characterized by a C6-C3-C6 backbone structure [8], with their classification based on the oxidation state of the central heterocyclic ring, including subclasses chalcones, flavonols, flavones, isoflavones, anthocyanidins, flavanones, and flavanols [9, 10]. Flavonoid biosynthesis initiates with chalcone synthase (CHS), which catalyzes the conversion of *p*-coumaroyl-CoA to naringenin chalcone—the committed step diverting phenylpropanoid precursors from lignin and hydroxycinnamic acid biosynthesis into flavonoid metabolism [11, 12]. Chalcone isomerase (CHI) then isomerizes naringenin chalcone to naringenin, which is hydroxylated by flavanone 3-hydroxylase (F3H) to form dihydrokaempferol. Flavonoid 3′-hydroxylase (F3′H) and flavonoid 3′,5′-hydroxylase (F3′5′H) convert dihydrokaempferol to dihydroquercetin and dihydromyricetin, respectively, thereby expanding the substrate pool for anthocyanin biosynthesis. These colorless dihydroflavonols are then transformed into colored anthocyanins through sequential actions of dihydroflavonol reductase (DFR) and anthocyanidin synthase (ANS), followed by a series of modifications including glycosylation, methylation, and acylation [13, 14]. Notably, naringenin and dihydrokaempferol are branching points in flavonoid metabolism, being alternatively channeled by flavone synthase (FNS) and flavonol synthase (FLS) to produce flavones and flavonols, respectively. This metabolic flexibility facilitates plants to dynamically regulate the production of distinct flavonoid subclasses from common precursors responding to developmental or environmental cues [15, 16].

Flavonoid biosynthesis is primarily controlled by MYB transcription factors (TFs), categorized by their conserved DNA-binding repeats (1R-4R) [17–21]. Notably, R2R3-MYBs (containing R2 and R3 repeats) serve as the dominant activators of flavonoid pathways in plants [19], and are subdivided into functionally distinct clades, that govern specific subclasses such as anthocyanins and flavonols [22]. Anthocyanin-activating R2R3-MYBs typically require specific bHLH cofactors and interact with WD40 proteins to form the MYB-bHLH-WD40 (MBW) complex, which specifically activates late biosynthesis genes (LBGs), such as *DFR* and *ANS*, directing flux toward anthocyanins [23, 24]. However, early biosynthesis genes (EBGs), such as *CHS*, *CHI*, *F3H,* and *FLS*, are usually controlled independently by MYB activators, particularly those governing flavonol production [25, 26]. While MYB activators drive flavonoid production, MYB repressors play an equally critical role in fine-tuning metabolic output to meet developmental needs and environmental challenges [27, 28]. For instance, the expression of *AtMYBL2*, an anthocyanin-repressing R3-MYB in *Arabidopsis thaliana*, is directly inhibited by two other repressors, AtHY5 and AtMYBD, whose expression is modulated by environmental factors including light and temperature [29]; meanwhile, AtHY5 directly targets the *AtMYBD* promoter to transcriptionally regulate its expression [30]. This hierarchical repression forms a sensitive control system that transduces environmental signals into precise anthocyanin regulation under stress conditions [31, 32].

Lilies (*Lilium* spp.) are significant ornamental crops, valued for its production of cut and potted flowers, as well as its ground-cover application in landscaping. However, lilies demonstrate a restricted adaptability to temperature, thriving within the range of 5°C–30°C. Furthermore, lilies are non-model species that exhibit gametophytic self-incompatibility, along with a complex genetic background and a highly heterozygous genome (33–36 Gb) [33, 34]. Thus, elucidating the molecular basis underlying cold stress tolerance in lilies holds both fundamental and practical significance [35]. Tiger lily (*Lilium lancifolium*) is one of the traditional lilies in China from section Sinomartagon with highly ornamental, edible, and medicinal values, which is a vital cross-breeding germplasm resource for the Asiatic hybrid lily cultivars [36]. Our preliminary genetic evaluation reveals that tiger lily possesses unique transcriptional regulatory mechanisms that confer its exceptional tolerance to extreme low temperatures (−35°C) [37]. Moreover, building on the co-expression network analysis of tiger lily cold-stressed transcriptomes [38], we characterized the hub gene *LlMYB3* as a positive regulator of cold tolerance, demonstrating its direct binding to the *LlCHS* promoter [39]. However, it remains unclear whether and how LlMYB3 mediates cold-induced or reduced flavonoid biosynthesis in tiger lily. Comprehensive sequence analysis in this study revealed that LlMYB3 belongs to the CPC-type R3-MYB, a subgroup of MYB TFs known for acting as anthocyanin repressors, and we thus redesignated LlMYB3 as LlR3MYB. Here, we propose a novel regulatory model in which the R3-MYB anthocyanin repressor LlR3MYB improves cold resistance via a flavonoid-mediated feedback loop in tiger lily.

## Results

### LlR3MYB belongs to anthocyanin R3-MYB repressors but positively associated with flavonoid biosynthesis

**Figure 1:**
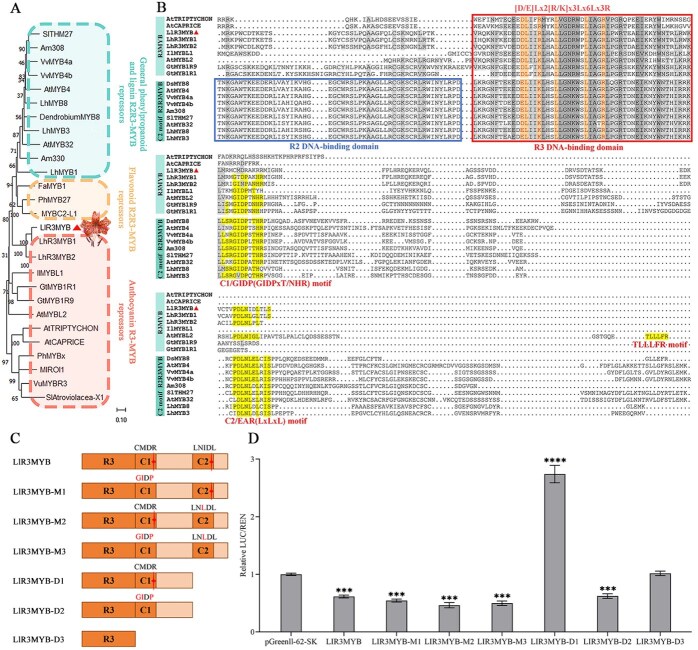
The LlR3MYB protein in tiger lily. (A) Phylogenetic analysis of general phenylpropanoid and lignin, flavonoid and anthocyanin repressed MYB genes from different plants. LlR3MYB are marked by the red triangle and flower image of tiger lily. (B) Amino acid sequence alignment of general phenylpropanoid and lignin, flavonoid and anthocyanin repressed MYB genes from different plants. LlR3MYB are marked by the red triangle. If more than 15 amino acids are the same, these amino acids are shown against yellow backgrounds. Horizontal lines represent the R2 and R3 repeat regions. The predicted amino acid sequences were obtained from the DDBJ/EMBL/GenBank database using the accession numbers listed in Table S2. (C) Structural schematics of LlR3MYB protein domains, deletion, and mutant constructs. The altered C1/C2 motif is marked with a red asterisk. (D) Transcriptional regulatory activity of LlR3MYB, deletions, and mutants in transient tobacco protoplast assay. BD vector is a negative control for transcriptional activation activity. Values are mean ± SD from three independent biological replicates (^***^P < 0.001; *t*-test).

LlR3MYB contained a 462-bp ORF, encoding 153 aa protein and belonging to the anthocyanin R3-MYB repressors, as shown in the phylogenetic tree constructed by known MYB repressors in general biosynthesis of phenylpropanoid and lignin, flavonoid biosynthesis, and anthocyanin biosynthesis (Fig. 1A). Amino acid sequence alignment of R3-MYB proteins showed that LlR3MYB shared 88% and 73% identity with LhR3MYB1 from Asiatic hybrid lily ‘Lollypop’ and LhR3MYB2 from Oriental hybrid lily ‘Dizzy,’ respectively (Fig. 1B). In the N-terminal region, LlR3MYB contained a conserved six-residue motif, [D/E]Lx2[R/K]x3Lx6Lx3R, required for interactions with R-like bHLH proteins in the R3 repeat, indicating LlR3MYB is a member of the CPC-type R3-MYB subgroup. To identify potential bHLH interaction partners, we conducted a Y2H screen against a tiger lily cDNA library using LlR3MYB as bait: the LlR3MYB protein demonstrated no self-activation activity (Fig. S1A); 56 positive clones were obtained that survived on selective medium through screening (Fig. S1B); sequencing revealed one clone carried a gene encoding an anthocyanin bHLH activator (UniProt: E3SXU4.1, Table S1); the physical interaction between LlR3MYB and this bHLH factor was confirmed by pairwise Y2H and BiFC assay (Fig. S1C and D). Furthermore, mutations in the major repression domains, the C1 and C2/EAR motifs, were found in the C-terminal region of LlR3MYB; these mutations involved a shift from [GIDP] to [CMDR] in the C1 motif and from [LxLxL] to [LxIxL] in the C2/EAR motif (Fig. 1B). Furthermore, we systematically assessed the transcriptional regulatory activity of full-length LlR3MYB, along with its C-terminal deletions and mutants carrying the canonical repressive C1 motif (GIDP), the C2/EAR motif (LNLDL), or both, using a GAL4-based reporter system in tobacco protoplasts (Fig. 1C). While both LlR3MYB and its mutant forms generally reduced reporter LUC activity, a distinct mutant lacking the C2/EAR-like motif (LNIDL) but containing a [CMDR] motif in place of the C1 motif (GIDP) exhibited a significant induction of LUC activity; in contrast, the simultaneous deletion of both [CMDR] and [LNIDL] motifs restored reporter activity to basal levels (Fig. 1D). These findings indicate that LlR3MYB possesses transcriptional repression activity that depends on the C2/EAR-like motif (LNIDL), while the [CMDR] motif may function as a potential activation domain.

**Figure 2:**
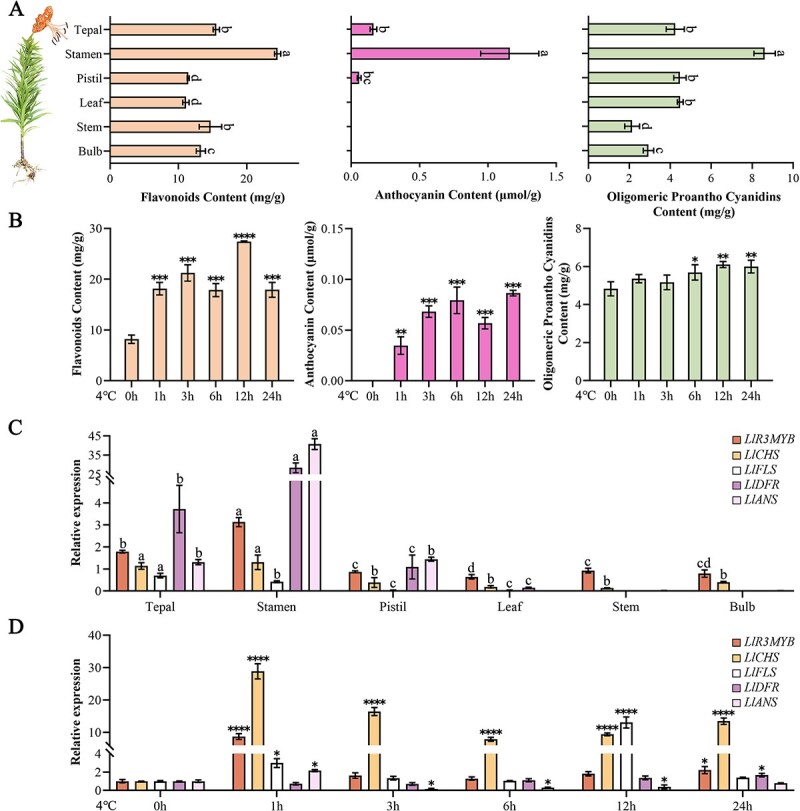
Spatiotemporal expression patterns of *LlR3MYB* and flavonoid accumulation in tiger lily. (A, B) Total contents of flavonoid, anthocyanin, and proanthocyanidin in different organs (A), and leaves at different times exposed to 4°C (B). (C, D) The relative expression of *LlR3MYB*, *LlCHS*, *LlFLS*, *LlDFR*, and *LlANS* in different organs (C), and leaves at different times exposed to 4°C (D). Values are mean ± SD from three independent biological replicates. Lowercase letters (a-c for A; a-d for C) indicate significant differences (*P* < 0.05, *t*-test). Asterisks denote ^*^P < 0.05, ^**^P < 0.01, ^***^P < 0.001,^****^P < 0.0001.

Spatiotemporal analysis revealed coordinated patterns between flavonoid accumulation and *LlR3MYB* expression. Under control conditions, stamens contained significantly higher flavonoid levels than bulbs, leaves, stems, tepals, and pistils (Fig. 2A). During cold treatment, leaf flavonoids increased rapidly within 1 h and remained elevated (Fig. 2B). Notably, *LlR3MYB* expression showed organ-specific correlation with flavonoids (Fig. 2C) and dynamic cold-induction: peaking at 1 h, declining (3–12 h), and recovering at 24 h (Fig. 2D). Meanwhile, the transcript profiles of key flavonoid enzyme genes (*LlCHS*, *LlFLS*, *LlDFR,* and *LlANS*) identified through co-expression network analysis of cold-stressed leaf transcriptomes of tiger lily were also examined, among which *LlCHS* exhibited an expression profile comparable to that of *LlR3MYB* (Fig. 2C and D). *LlCHS* was highly induced within 1 h of 4°C treatment with a 29-fold increase compared to the control (Fig. 2D). Therefore, *LlR3MYB* and *LlCHS* might contribute to the flavonoid biosynthesis in tiger lily, particularly under cold stress. Although stamens accumulated relatively high levels of anthocyanins and proanthocyanidins (Fig. 2A), consistent with the elevated expression of *LlDFR* and *LlANS* (Fig. 2C), the dynamic change of these metabolites under cold stress (Fig. 2B) did not correlate with the transcription patterns of these genes (Fig. 2D). This suggests that transcriptional activation may not be responsible for the rapid fine-tuning of anthocyanin and proanthocyanidin levels in response to cold.

### Ectopic expression of *LlR3MYB* enhanced flavonoid production and cold tolerance in transgenic tobacco

**Figure 3:**
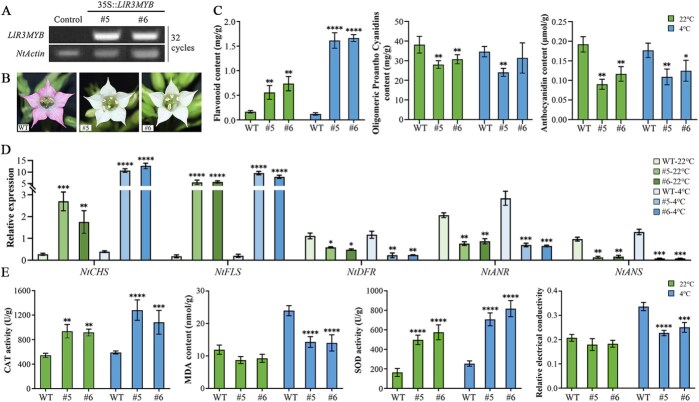
Physiological and molecular characterization of cold stress responses in *LlR3MYB*-overexpressing transgenic tobacco plants. (A) Semi-quantitative RT-PCR validation of *LlR3MYB* in transgenic lines (#5, #6). (B) Flower tepal pigmentation comparison between wild-type (WT, pink) and transgenic (white) plants. (C) Quantification of total flavonoids, proanthocyanidins, and anthocyanins in WT and transgenic tobacco under control (22°C) and cold (4°C) conditions. (D) qRT-PCR analysis of flavonoid pathway gene expression in WT and transgenic tobacco under control (22°C) and cold (4°C) conditions. (E) Quantification of SOD and CAT activity, MDA content and REC in WT and transgenic tobacco under control (22°C) and cold (4°C) conditions. Values are mean ± SD from three independent biological replicates (^*^P < 0.05; ^**^P < 0.01; ^***^P < 0.001; ^****^P < 0.0001; *t*-test).

Overexpression of *LlR3MYB* in tobacco (35S::*LlR3MYB*) was confirmed by RT-PCR (Fig. S2A). Transgenic lines #5 and #6 exhibited the highest transgene expression and were further characterized (Fig. 3A). Phenotypic analysis revealed that transgenic tobacco lines were morphologically indistinguishable from wild-type (WT) plants except for their floral pigmentation. While WT plants developed characteristic pink tepals, transgenic lines produced white tepals (Fig. 3B). This visual difference correlated with significantly lower anthocyanin accumulation in transgenic tepals both before and after 4°C treatment (Fig. S2B). In leaves, transgenic plants consistently showed 3.4- to 13.7-fold higher flavonoid levels, and 9 to 53% reduced proanthocyanidin and anthocyanin content compared to WT under both control (22°C) and cold stress (4°C) conditions (Fig. 3C). qRT-PCR analysis revealed that *LlR3MYB* overexpression upregulated *NtCHS* and *NtFLS*, exhibiting an over 10-fold increase than WT after 4°C treatment, whereas downregulating core anthocyanin pathway genes (*NtDFR*, *NtANS,* and *NtANR*) (Fig. 3D). Notably, flavonoid and proanthocyanidin accumulation and upregulation of *NtCHS*, *NtFLS*, and *NtANR* were only triggered after 2 h at 4°C in transgenic tobacco flowers (Fig. S2C). Furthermore, transgenic plants exhibited enhanced cold tolerance, as evidenced by significantly elevated SOD/CAT activities and reduced MDA/electrolyte leakage following cold treatment (Fig. 3E).

### Transcriptome, proteome, and metabolome analysis of *LlR3MYB*-overexpressed tiger lily

**Figure 4:**
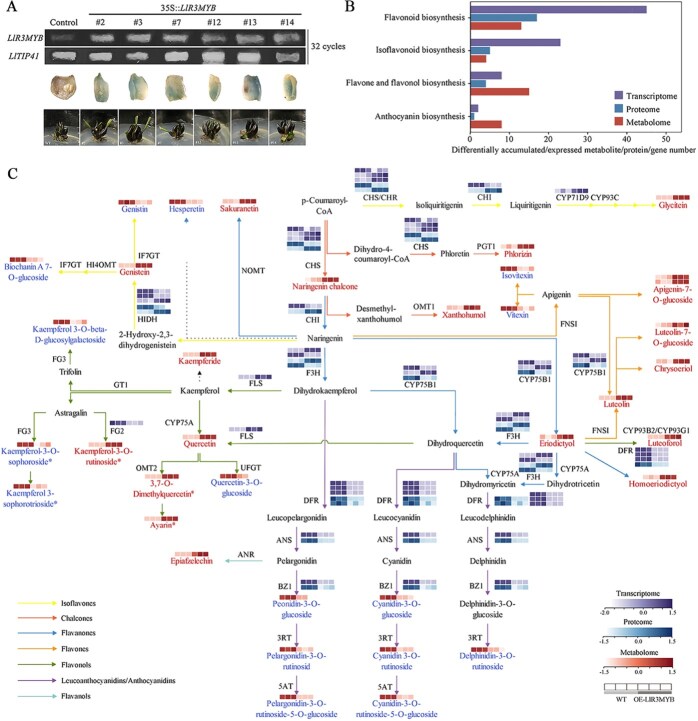
Phenotypes and multi-omics analysis of transgenic tiger lily harboring *LlR3MYB* gene. (A) Semi-quantitative RT-PCR analysis, GUS staining and morphological characteristics of the *LlR3MYB-*overexpressing plants (OE-LlR3MYB) and wild type (WT). (B) KEGG enrichment of DEGs, DEPs, and DAMs. (C) Multi-omics integrative analysis of flavonoid biosynthesis in OE-LlR3MYB and WT. The names of the upregulated and downregulated DAMs were typed in red and blue, respectively. Enzyme names are detailed in Table S6.

To further elucidate the comprehensive biochemical and molecular functions of *LlR3MYB*, we employed RNA-seq, UHPLC–MS/MS, and UPLC-ESI-MS/MS system to analyze the transcriptomic, proteomic, and metabolomic profiles of tiger lily plants overexpressing *LlR3MYB*. Overexpression of *LlR3MYB* in tiger lily was verified by RT-PCR and GUS activity staining (Fig. 4A). The regenerated plants of *LlR3MYB*-overexpressing tiger lily (OE-LlR3MYB) displayed no difference from WT in terms of morphological characteristics (Fig. 4A). However, principal component analysis (PCA) revealed that OE-LlR3MYB and WT samples were grouped or discriminated from each other, indicating significant differences in their transcriptomic, proteomic, and metabolomic profiles (Fig. S3A).

Transcriptomic profiling identified 2816 upregulated and 2826 downregulated differentially expressed genes (DEGs) in OE-LlR3MYB compared with WT (Table S3). KEGG analysis indicated 2875 metabolism-related DEGs (Fig. S3B), among which 45, 23, 8, and 2 DEGs were relevant to flavonoid, isoflavonoid, flavone and flavonol, and anthocyanin biosynthesis, respectively (Fig. 4B, Table S4). Proteomic analysis quantified 6754 proteins, with 443 upregulated and 430 downregulated differentially expressed proteins (DEPs) (Table S5). KEGG analysis revealed 457 metabolism-related DEPs which showed similar pathway enrichment to transcriptome (Fig. S3C), including 17, 5, 4, and 1 DEPs involved in flavonoid, isoflavonoid, flavone and flavonol, and anthocyanin biosynthesis, respectively (Fig. 4B, Table S4). In terms of metabolomic profiles, the total ion current (TIC) overlap analysis validated sample quality and analytical consistency (Fig. S3D). In the KEGG database, 567 ions were classified as distinct metabolites, primarily associated with 6 KEGG pathways (Fig. S3E), with 96.83% identified as flavonoids and 3.17% as tannins. Among these, 170 upregulated and 101 downregulated differentially accumulated metabolites (DAMs) were detected (Table S6), with 13, 4, 15, and 8 DAMs linked to flavonoid, isoflavonoid, flavone and flavonol, and anthocyanin biosynthesis, respectively (Fig. 4B, Table S4).

Integrated analysis of DEGs, DEPs, and DAMs annotated in the KEGG flavonoid biosynthesis pathway (Fig. 4C, Table S7) demonstrated that *LlR3MYB* overexpression preferentially activates flavonol biosynthesis while repressing anthocyanidin production. The biosynthesis of flavonols was highly activated in OE-LlR3MYB, as evidenced by the 3.99-, 3.33-, 3.15-, 2.06-, and 1.73-fold increases in the accumulation of 3,7-O-dimethylquercetin, ayarin, kaempferide, quercetin and kaempferol-3-O-rutinoside; besides, significant upregulation of flavones (luteolin 7-O-glucuronide, apigenin-7-O-glucoside, luteolin and chrysoeriol) and chalcones (naringenin chalcone, xanthohumol, and phlorizin) were also observed; similarly, the contents of isoflavones (i.e. genistein and glycitein), flavanones (i.e. homoeriodictyol, sakuranetin, and eriodictyol), and flavanols (i.e. luteoforol and epiafzelechin) also increased. In contrast, the biosynthesis of anthocyanidins was highly repressed in OE-LlR3MYB, as evidenced by the 3.11-, 3.02-, 2.65-, 2.19-, 2.13-, 1.33-, and 1.11-fold decreases in the accumulation of pelargonidin-3-O-rutinoside-5-O-glucoside, cyanidin-3-O-rutinoside-5-O-glucoside, delphinidin-3-O-rutinoside, cyanidin 3-O-rutinoside, cyanidin 3-O-glucoside, peonidin-3-O-glucoside, and pelargonidin-3-O-rutinoside. In accordance with DAM profiles, LlCHS and LlF3H showed significantly increased expression in OE-LlR3MYB at both transcriptome and proteome levels, as well as LlFLS (transcriptional upregulation) and LlCHI (protein-level upregulation) were enhanced; while LlDFR, LlANS, and anthocyanidin 3-O-glucosyltransferase (LlBZ1) were suppressed at both transcriptome and proteome levels.

### Silence of *LlR3MYB* affected low-temperature oxidation resistance and flavonoid biosynthesis in tiger lily

**Figure 5:**
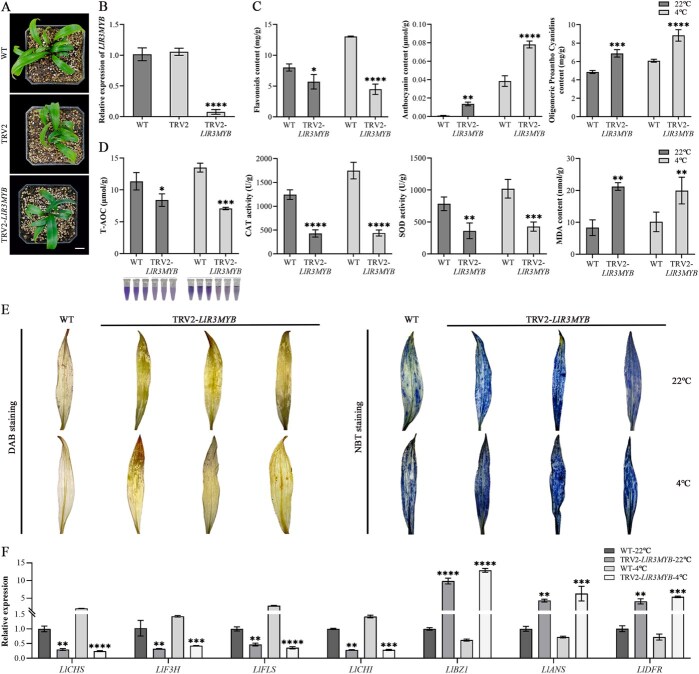
Cold stress response phenotypes of *LlR3MYB*-silenced tiger lily plants. (A) Representative phenotype of TRV2-*LlR3MYB* transgenic plants compared to the controls. Scale bar = 2 cm. (B) Validation of *LlR3MYB* silencing by qRT-PCR. (C) Quantification of total flavonoids, proanthocyanidins, and anthocyanins in WT and transgenic plants under control (22°C) and cold (4°C) conditions. (D, E) Biochemical analyses of (D) antioxidant capacity (T-AOC, SOD, CAT activity), and (E) oxidative stress markers (MDA levels, DAB and NBT staining) in WT and transgenic plants under control (22°C) and cold (4°C) conditions. (F) qRT-PCR analysis of key flavonoid pathway gene expression in WT and transgenic plants under control (22°C) and cold (4°C) conditions. Values are mean ± SD from three independent biological replicates (^*^P < 0.05; ^**^P < 0.01; ^***^P < 0.001; ^****^P < 0.0001; *t*-test).

To further analyze the function of *LlR3MYB*, a TRV2-*LlR3MYB* construct was transiently expressed in tiger lily cutting seedlings and flower tepals. The regenerated plants of *LlR3MYB*-silencing tiger lily (TRV2-*LlR3MYB*) cutting seedlings displayed no difference from WT in terms of morphological characteristics (Fig. 5A). The instantaneous silence of *LlR3MYB* in TRV2-*LlR3MYB*-infiltrated cutting seedlings was verified by qRT-PCR (Fig. 5B). The TRV2-*LlR3MYB* showed a significant decrease in total flavonoids, coupled with marked increases in both proanthocyanidins and anthocyanins, relative to the WT both before and after 4°C treatment (Fig. 5C). Consequently, a reduced total antioxidant capacity (T-AOC), as well as lower levels of SOD and CAT activities, were observed in TRV2-*LlR3MYB* (Fig. 5D). Moreover, given that the T-AOC was assessed using the FRAP (Ferric Reducing Ability of Plasma) method, the lighter blue color observed in the TRV2-*LlR3MYB* samples (Fig. 5D) suggests a decreased production of the blue compound Fe^2+^-TPTZ [40]; this observation also confirmed a reduction in T-AOC for TRV2-*LlR3MYB* compared to the WT. While 4°C treatment increased MDA levels in both transgenic and WT plants, the transgenic lines accumulated significantly higher MDA concentrations (Fig. 5D). Similarly, stronger DAB and NBT staining was observed in TRV2-*LlR3MYB* transgenic plants both before and after 4°C treatment (Fig. 5E). Besides, the instantaneous silence of *LlR3MYB* significantly down-regulated the expression of *LlCHS*, *LlF3H*, *LlFLS,* and *LlCHI*, whereas the expression of *LlDFR*, *LlANS,* and *LlBZ1* were up-regulated in TRV2-*LlR3MYB* (Fig. 5F).

**Figure 6:**
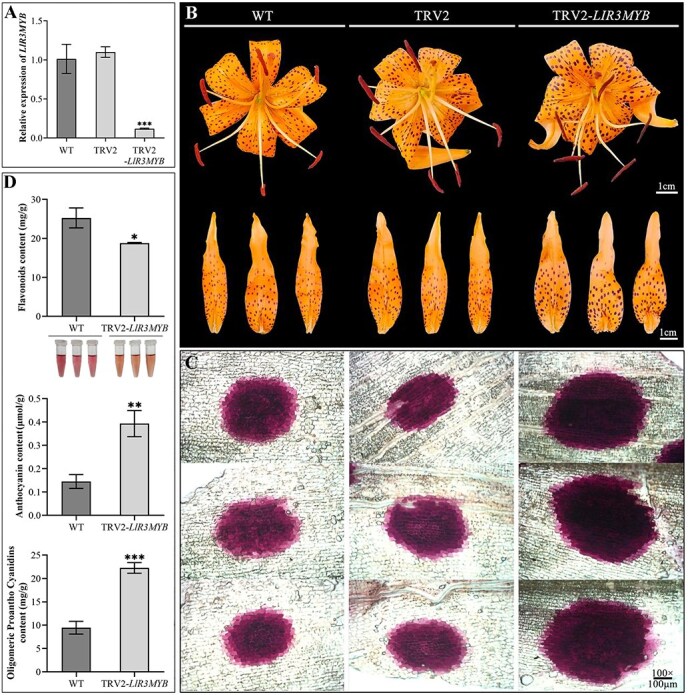
Phenotypes of *LlR3MYB*-silenced tiger lily tepals. (A) Validation of *LlR3MYB* silencing by qRT-PCR. (B) Representative phenotype of TRV2-*LlR3MYB* transgenic tepals compared to the controls. Scale bar = 1 cm. (C) Comparison of pigmented deposits in the upper epidermal cells of WT, TRV2, and TRV2-*LlR3MYB* tepals. The total magnification is 100 ×. Scale bar = 100 μm. (D) Quantification of total flavonoids, anthocyanins, and proanthocyanidins in WT and TRV2-*LlR3MYB* transgenic tepals. Values are mean ± SD from three independent biological replicates (^*^P < 0.05; ^**^P < 0.01; ^***^P < 0.001; ^****^P < 0.0001; *t*-test).

Following the successful silencing of *LlR3MYB* (confirmed via qRT-PCR, Fig. 6A), TRV2-*LlR3MYB*-infiltrated flower tepals exhibited a phenotype of significantly enlarged spots (Fig. 6B). Freehand sectioning further revealed a greater number of cells with more intensely pigmented deposits in the upper epidermis of TRV2-*LlR3MYB* compared to WT (Fig. 6C). Consistent with this observation, anthocyanin and proanthocyanidin contents were significantly elevated, while the total flavonoid level was markedly reduced in the TRV2-*LlR3MYB* tepals (Fig. 6D).

### LlR3MYB directly binds to the AC-I and MBSI element in the *LlCHS2* promoter and activates its transcription

Transcriptomic and proteomic analyses revealed significant upregulation of chalcone synthase (LlCHS2, Cluster_50882), the gateway enzyme of flavonoid biosynthesis, in *LlR3MYB*-overexpressing tiger lily. Sequence analysis revealed the LlCHS2 (GenBank accession number: PV761029.1) coding sequence (1179 bp) encodes a 392-amino acid protein that shows high conservation with known chalcone synthases [41] (Fig. S4). More importantly, alignment of LlCHS2 with the previously identified LlCHS (co-expressed with LlR3MYB in Fig. 2) demonstrated significant sequence homology.

**Figure 7:**
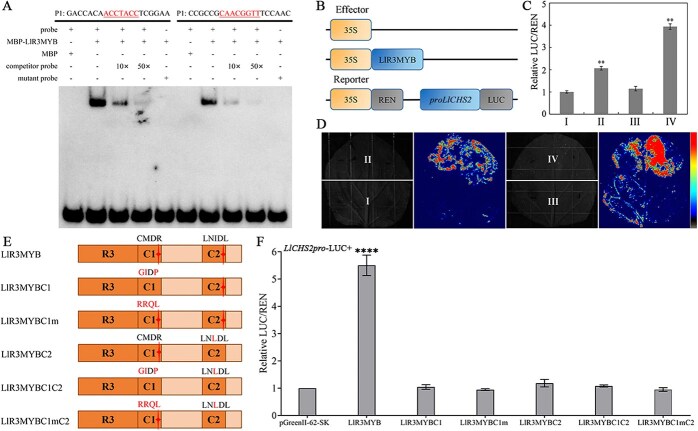
Molecular mechanism of LlR3MYB-mediated activation of *LlCHS2* expression. (A) EMSA of LlR3MYB binding to the AC-I element (ACCTACC) (P1) and MBSI motif (CAACGGTT) (P2) in the promoter of *LlCHS2*. Competitors in 10-fold or 50-fold molar excess relative to the labeled probes were included. (B) Schematic of dual-luciferase assay constructs (effector: LlR3MYB; reporter: *LlCHS2*pro-LUC). (C, D) The relative LUC/REN ratio (C) and bright/dark field images (D) of tobacco leaves in the transient expression assays. I: pGreenII-62-SK and *LlCHS2*pro-0800-LUC (25 ± 2°C); II: LlR3MYB-62-SK and *LlCHS2*pro-0800-LUC (25 ± 2°C); III: pGreenII-62-SK and *LlCHS2*pro-0800-LUC (after 4°C for 24 h); IV: LlR3MYB-62-SK and *LlCHS2*pro-0800-LUC (after 4°C for 24 h). (E) Structural schematics of LlR3MYB protein domains and mutant constructs. The altered C1/C2 motif is marked with a red asterisk. (F) The relative LUC/REN ratio of tobacco leaves in the transient expression assays after 4°C for 24 h (effector: LlR3MYB and its mutant variants; reporter: *LlCHS2*pro-LUC). Values are mean ± SD from three independent biological replicates (^**^P < 0.01; ^****^P < 0.0001; *t*-test).

Based on these findings and the observed interaction of LlR3MYB and *LlCHS2* promoter [39], we employed EMSA (electrophoretic mobility shift assay) to characterize the direct interaction between LlR3MYB and the *LlCHS2* cis-elements. As shown in Fig. 7A, slower migrating bands were detected when the LlR3MYB-MBP fusion protein was incubated with the AC-I element (ACCTACC) and MBSI motif (CAACGGTT). Furthermore, a more distinct band was detected in the presence of AC-I element biotin probe and the band was abolished by the presence of 50-fold cold competitive probe, which implies LlR3MYB had higher affinity for AC-I element than MBSI in the *LlCHS* promoter. Dual luciferase assays revealed LlR3MYB-mediated transcriptional activation of the *LlCHS2* promoter, with the experimental design (Fig. 7B) and corresponding quantitative results (Fig. 7C) showing 2-fold (25°C) and 4-fold (4°C) increases in LUC/REN ratios compared to controls. The corresponding results were observed in LUC signal bioluminescence (Fig. 7D).

Considering the C-terminal C1 (GIDP) and C2 (LNLDL) motifs are known to confer repressive function in MYB repressors [42], we generated five LlR3MYB mutants designated as LlR3MYBC1 (GIDP), LlR3MYBC1m (RRQL), LlR3MYBC2 (LNLDL), LlR3MYBC1C2 (GIDP+LNLDL), and LlR3MYBC1mC2 (RRQL+LNLDL), respectively, to assess their impact on the transcriptional activation of the *LlCHS2* promoter (Fig. 7E). Dual luciferase assays revealed that these LlR3MYB mutants either lost or showed significantly reduced activation of the *LlCHS2* promoter (Fig. 7F).

### LlDREB binds to the *LlR3MYB* promoter and activates its transcription in a low-temperature dependent manner

**Figure 8:**
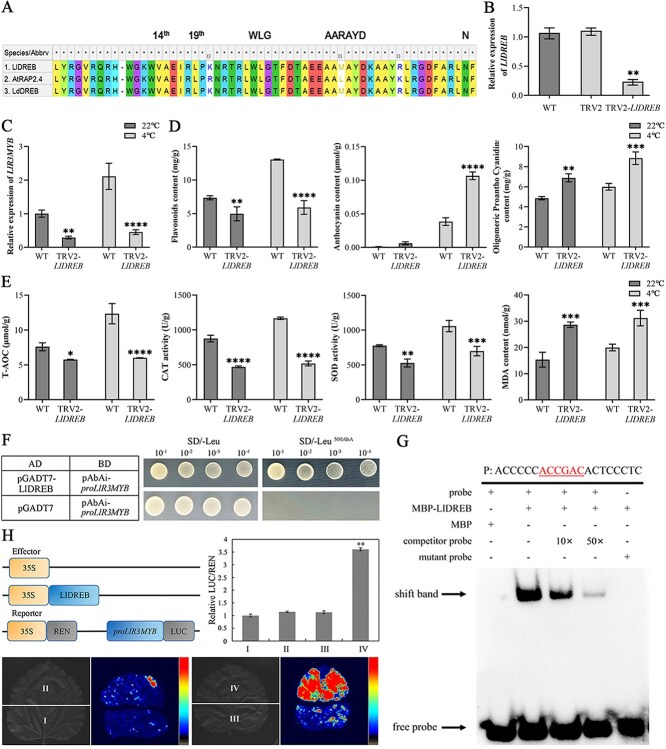
Cold-dependent transcriptional activation of *LlR3MYB* by LlDREB. (A) The amino acid sequence alignment for the LlDREB was conducted in comparison to the *A. thaliana* AtRAP2.4 (AT1G78080), *Lilium davidii* var. *unicolor* LdDREB (AMA66330.1). (B) Validation of *LlDREB* silencing in tiger lily plants by qRT-PCR. (C) qRT-PCR analysis of *LlR3MYB* gene expression in WT and transgenic plants under control (22°C) and cold (4°C) conditions. (D) Quantification of total flavonoids, proanthocyanidins, and anthocyanins in WT and transgenic plants under control (22°C) and cold (4°C) conditions. (E) Biochemical analyses of (D) antioxidant capacity (T-AOC, SOD, CAT activity), and MDA levels in WT and transgenic plants under control (22°C) and cold (4°C) conditions. (F) Y1H validation of LlDREB binding to the *LlR3MYB* promoter. Yeast strain Y1HGold co-transformed with experimental group (pGADT7-LlDREB and pAbAi-pro*LlR3MYB*) and negative control (pGADT7 and pAbAi-pro*LlR3MYB*) were plated on selective media: SD/-Leu and SD/-Leu/-500 ng/ml AbA. (G) EMSA of LlDREB binding to the DRE (ACCGAC) motif (P) in the promoter of *LlR3MYB*. Competitors in 10-fold or 50-fold molar excess relative to the labeled probes were included. (H) The relative LUC/REN ratio and bright/dark field images of tobacco leaves in the transient expression assays. I: pGreenII-62-SK and *LlR3MYB*pro-0800-LUC (25 ± 2°C); II: LlDREB-62-SK and *LlR3MYB*pro-0800-LUC (25 ± 2°C); III: pGreenII-62-SK and *LlR3MYB*pro-0800-LUC (after 4°C for 24 h); IV: LlDREB-62-SK and *LlR3MYB*pro-0800-LUC (after 4°C for 24 h). Values are mean ± SD from three independent biological replicates (^**^P < 0.01; ^***^P < 0.001; ^****^P < 0.0001; *t*-test).

To identify upstream regulators of *LlR3MYB*, we isolated a 1374 bp promoter fragment (Table S8) and cloned it into the pBait-AbAi vector for Y1H screening. The cDNA library was constructed from cold-treated (4°C, 2 h) tiger lily tepals and leaves to capture cold stress-responsive factors. We identified 22 clones that survived on selective medium (Fig. S5), including one clone that carried the gene encoding a dehydration responsive element (DRE) binding factor DREB (Table S9). The coding sequence of LlDREB was 915 bp in length encoding 304 amino acids, which was identical to the cold-responsive LlDREB (AHK24883.1) previously identified in tiger lily [37]. Phylogenetic analysis showed that LlDREB clustered in the *A. thaliana* subgroup A-6 DREBs (Fig. S6). Multiple sequence alignment showed that LlDREB shared 95% and 46% identity with LdDREB (AMA66330.1) of *Lilium davidii* var. *unicolor*, and AtRAP2.4 (AT1G78080) of *A. thaliana.* All three proteins share the WLG motif and invariant V14 and L19 in the AP2 domain (Fig. 8A), suggesting functional maintenance within the clade.

To elucidate the function of LlDREB, tiger lily cutting seedlings were transiently transformed with a TRV2-*LlDREB* construct, as confirmed by qRT-PCR (Fig. 8B). TRV2-*LlDREB* plants exhibited significantly downregulated *LlR3MYB* expression (Fig. 8C), along with decreased total flavonoid content and elevated accumulation of proanthocyanidins and anthocyanins compared to the WT (Fig. 8D), an effect that was more pronounced following 4°C treatment. Concomitantly, these plants displayed decreased T-AOC, lower activities of the antioxidant enzymes SOD and CAT, and higher MDA concentrations (Fig. 8E). In contrast, opposite phenotypic trends were obtained in *LlDREB-*overexpressing lilies (Fig. S7), supporting a positive role for LlDREB in mitigating oxidative stress under cold conditions.

Bioinformatic analysis identified a DRE (ACCGAC) motif in the *LlR3MYB* promoter region (Table S8). Y1H assays demonstrated direct binding of LlDREB to this promoter, as evidenced by growth of co-transformed yeast with pAbAi-pro*LlR3MYB* and pGADT7-LlDREB on selective medium containing 500 ng/ml AbA, while control transformants failed to grow (Fig. 8F). We then performed an EMSA with an MBP-LlDREB fusion protein. Incubation with a labeled DRE motif (ACCGAC) probe resulted in a distinct band shift (Fig. 8G). Dual-luciferase reporter assays revealed low temperature-dependent transcriptional regulation of *LlR3MYB* driven by LlDREB: although LlDREB showed minimal effect at 25°C, it induced a 3.6-fold increase in *LlR3MYB* promoter activity following 24-h cold treatment (4°C); the corresponding results were observed in LUC signal bioluminescence (Fig. 8H). These findings establish that LlDREB specifically binds and activates the *LlR3MYB* promoter in a cold-responsive manner.

## Discussion

Cold stress severely constrains plant growth and development, posing significant challenges to horticultural production. As a commercially important ornamental, lily is also vulnerable to cold stress damage. At the cellular level, cold stress triggers ROS imbalance [43–45]; in return, induced flavonoid production contributes to the elimination of excess ROS and improved cold tolerance [1, 46–49]. Our previous transcriptome analysis of cold-stressed tiger lily identified a R3-MYB TF LlR3MYB that was characterized as positively regulating cold resistance and directly binding to the *LlCHS* promoter. Given that multiple MYBs play crucial roles in regulating both cold stress response and flavonoid accumulation [22, 27, 50, 51], we further investigated the connections between LlR3MYB, flavonoid accumulation, and cold stress responses.

Comprehensive sequence analysis in this study revealed that LlR3MYB belongs to the CPC-type R3-MYB, a subgroup of MYB TFs known for acting as anthocyanin repressors (Fig. 1). Mechanistically, CPC-type R3-MYB TFs repress late biosynthesis genes (LBGs) by competitively disrupting the interaction between anthocyanin-activating R2R3-MYBs and their bHLH cofactors, thereby preventing transcriptional activation of the anthocyanin pathway [52, 53]. This mechanism has been demonstrated in LhR3MYB2 and LhR3MYB15 in Asiatic hybrid lilies [54], AtMYBL2 [29] and AtCAPRICE [55] in Arabidopsis, MlROI1 in *Mimulus* [56], GtMYB1R1 and GtMYB1R9 in gentian [57], SlMYBATV in tomato [58], IlMYBL1 in *Iochroma* [59], and PtMYB182 in poplar [60]. In tiger lily, we observed that LlR3MYB interacts with an anthocyanin biosynthesis bHLH activator (Fig. S1). Ectopic overexpression of *LlR3MYB* resulted in a color shift from pink to white in tobacco transformants, which was accompanied by reduced expression levels of *NtDFR* and *NtANS* (Fig. S2). Furthermore, significant decreases in anthocyanidin contents were observed in *LlR3MYB*-overexpressed tiger lilies, along with decreased expression levels of *LlDFR*, *LlANS*, and *LlBZ1* (Fig. 4); while increased expression levels of *LlDFR*, *LlANS*, and *LlBZ1* were observed in *LlR3MYB*-silenced tiger lilies (Fig. 5). Therefore, we propose that LlR3MYB functions as a typical anthocyanin repressor, suppressing the expression of the LBGs (i.e. *LlDFR*, *LlANS,* and *LlBZ1*) by competitively interfering with the R2R3MYB-bHLH activator complex formation. Furthermore, *LlR3MYB* shows preferential expression in pigmented floral organs under normal conditions (Fig. 2), as seen with LhR3MYB2 in Asiatic hybrid lilies [54], and PhMYBx in petunia [61, 62], which appears to function as a negative regulator of floral anthocyanin biosynthesis. Notably, the basal pigmentation of tiger lily tepals is primarily attributed to carotenoids, whereas the spotted pigmentation on the tepals results from anthocyanin deposition [63]. This pigment composition is consistent with our functional characterization of *LlR3MYB* silencing, which in flowers significantly increased anthocyanin content in the spotted regions while reducing total flavonoid levels (Fig. 6).

However, in alignment with our initial hypothesis, we found that LlR3MYB also positively regulates cold stress-induced flavonoid biosynthesis, revealing its dual regulatory function. In tiger lily, *LlR3MYB* was highly induced in leaves within 1 h of 4°C treatment and attained the highest level; similarly, flavonoid content were stimulated by 4°C treatment within 1 h and maintained high levels over the 4°C treatment duration (Fig. 2). Furthermore, there was a notable increase in flavonoid levels in *LlR3MYB-*overexpressed tobacco and tiger lily plants, along with increased expression levels of the early biosynthesis genes (EBGs), e.g. *LlCHS*, *LlF3H*, *LlFLS*, and *LlCHI*, particularly under 4°C treatment (Figs 3 and 4). Notably, it was found that flavonols, including 3,7-O-dimethylquercetin, ayarin, kaempferide, quercetin, and kaempferol-3-O-rutinoside, are the main flavonoids that are positively regulated by *LlR3MYB* (Fig. 4). A conserved stress response across land plants involves the preferential accumulation of quercetin (Que) derivatives over kaempferol (Kae), correlating with increasing organismal complexity [64–67]. Flavonoid-mediated ROS scavenging requires a B-ring catechol group, with optimal activity depending on C-ring structural features (C2-C3 unsaturation and 4-oxo group), as demonstrated in Que [68, 69]. While glycosylation enhances flavonoid water solubility, stabilizes against oxidation, and mediates ER-to-organelle transport, it partially compromises their antioxidant activity [7]. Consistent with structure–activity relationships, Que 3-O-glucoside shows intermediate antioxidant activity: lower than Que but higher than Kae, while Kae 3-O-glucoside is nearly inactive [7]. Given that *LlR3MYB* silencing in tiger lily reduced total flavonoid content, impaired antioxidant systems, and exacerbated oxidative stress (Fig. 5), we suggest LlR3MYB promotes cold tolerance by upregulating high-efficiency ROS scavengers (Que and Kae derivatives) while downregulating energetically costly antioxidants (anthocyanins including pelargonidin, cyanidin, and delphinidin glycosides). Despite the observed increase in total anthocyanins and proanthocyanidins under cold stress in WT tiger lily (Fig. 2B), this finding does not contradict but rather reinforces the role of R3-MYB proteins as anthocyanin negative regulators. Their function is to fine-tune, rather than completely shut down, anthocyanin biosynthesis to prevent over-accumulation, even under strongly inductive conditions [54].

Functional specialization and adaptive regulation of plant MYBs within a family, whose members exhibit substantial diversity in their roles, are primarily attributed to the C-terminal motifs [70]. The observed dual functionality of LlR3MYB likely stems from the structural configuration of its C-terminal motifs. Unlike typical R3-MYBs, which act as passive repressors lacking defined repression or activation domains [18, 19, 28], LlR3MYB harbors a C-terminal C2/EAR-like [LNIDL] repressor motif and a divergent C1 motif [CMDR] that exhibits potential transcriptional activation (Fig. 1). A similar coexistence of activation and repression motifs at the C-terminus has been reported in PvMYB4 [71], underscoring the structural complexity of LlR3MYB and its capacity for dual regulatory roles within sophisticated transcriptional networks. The C2/EAR (LxLxL) motif represents the most widely conserved transcriptional repression motif in plants, operating mainly through chromatin modification or direct interference with the basal transcriptional machinery [72–74]. Apart from the C2/EAR motif, nearly all R2R3-MYB repressors belong to subgroup 4 are also defined by a conserved C1 (GIDP) motif in the C-terminal region [75, 76]. However, the C1 motif displays less functional conservation among transcriptional repressors than the C2/EAR motif. For example, in Arabidopsis, the C1 motif of PvMYB4 acts as a potential activation domain [71], whereas in peach, the corresponding motif in PpMYB18 serves as a repression domain [42]. In the present study, the modified C1 motif [CMDR] conferred activation, whereas restoration of the canonical C1 (GIDP) motif restored repression (Fig. 1D). Notably, in peach PpMYB18, repression is mediated by both a bHLH-interaction motif in the R3 domain and the C-terminal C1/C2 motifs [42], further highlighting the mechanistic versatility of MYB repressors.

Interestingly, although LlR3MYB exhibits transcriptional repression dependent on the C2/EAR-like (LNIDL) motif in tobacco protoplasts, as shown in a GAL4-based reporter assay (Fig. 1D), it can bind directly to the AC-I element (ACCTACC, preferred) and the MBSI motif (CAACGGTT) in the *LlCHS2* promoter and activate its transcription, as demonstrated by EMSA and dual-luciferase assays (Fig. 7). A comparable regulatory pattern is seen in soybean HSF2B, which possesses repression activity and represses the flavonoid synthesis repressor *NAC* gene while directly activating flavonoid biosynthetic enzyme genes, thereby collectively enhancing flavonoid accumulation under salt stress [77]. The basis for such dual functionality remains incompletely understood but may involve interactions with transcriptional activators such as HSFA1a [77, 78]. In this study, mutation of the altered C1 (CMDR) motif or restore the canonical C2/EAR (LNLDL) motif in LlR3MYB renders the activator of the *LlCHS2* promoter inactive (Fig. 7F). These results imply that whereas the canonical C2/EAR (LxLxL) motif confers stable repression, the C2/EAR-like (LNIDL) motif fails to repress the *LlCHS2* promoter, thereby permitting the CMDR motif to exert its activation function. This functional divergence may be linked to post-translational modifications, such as phosphorylation and ubiquitination, which are known to modulate C2/EAR-mediated gene regulation positively or negatively by affecting the conformation, interaction partners, subcellular localization, and stability of the repressors or associated co-repressors [72]. The mutation or deletion of C2/EAR motifs in certain MYB repressors was also shown to compromise its repressor function. For instance, the deletion of the C2 motif in PvMYB4 abolishes its repressor function [71], while point mutations in the corresponding motif of MtMYB2 significantly impair transcriptional repression activity [79]. Slightly altered C2 motifs in poplar PtMYB182 (IxIxL) and AtMYBL2 (LxIxL) fail to confer repressive activity [29, 60]. Additionally, in transgenic apple callus, MdMYB16 fails to suppress anthocyanin accumulation when the C2 motif is absent [80]. Therefore, LlR3MYB was identified as the first R3MYB activator for *LlCHS2* expression, in contrast to the previous knowledge that the small R3MYB usually acts as repressor.

Cold stress (4°C) triggered biphasic *LlR3MYB* induction (1 h and 24 h; Fig. 2D), suggesting dual roles in responding to immediate chilling and later stress signaling. Y1H assay performed in this study demonstrated that LlDREB, a member of the DREB subfamily A-6, binds to the promoter region of *LlR3MYB* (Fig. 8A, F and Fig. S6). Further functional analysis indicated that LlDREB positively contributes to alleviating oxidative stress under cold conditions (Fig. 8C, D and Fig. S7). Similar to the well-characterized stress-responsive A-1 DREB1s/CBFs and A-2 DREB2s, A-6 DREBs also play a role in stress responses, albeit through regulation of distinct target genes [81]. In Arabidopsis A-6 DREBs, AtRAP2.4 responds to multiple stresses (dehydration, salinity, cold, and heat) and recognizes both DRE/CRT and GCC-box cis-elements [82, 83]. We identified a DRE core motif (ACCGAC) within the *LlR3MYB* promoter region that is bound by the LlDREB protein (Fig. 8G, Table S8). Interestingly, LUC reporter assays demonstrated temperature-dependent LlDREB activation of the *LlR3MYB* promoter, with significant signal enhancement exclusively under cold conditions (Fig. 8H). Building on the established redox regulation of *AtRAP2.4a* [84], we hypothesize that LlDREB’s transcriptional activation and DNA-binding affinity are similarly regulated by cold-induced alterations in cellular redox homeostasis. Combined with the rapid accumulation of flavonoids observed within 1 h of cold treatment (Fig. 2B), our results indicate the presence of regulatory mechanisms that extend beyond transcriptional activation. On one hand, the cold-induced upregulation of LlR3MYB (mediated by LlDREB) and its downstream target *LlCHS2* appears to be integrated into a broader epigenetic-transcriptional network, potentially modulated by dynamic histone modifications, chromatin reorganization, and enhancer activity [85–89]. On the other hand, the swift flavonoid response may be attributed to the direct post-translational activation (e.g. through allosteric effects or phosphorylation) of pre-existing flavonoid biosynthetic enzymes by cold stress [1, 90–92]. Collectively, these mechanisms suggest a multi-layered regulatory strategy for rapid and fine-tuned flavonoid production in tiger lily, the precise contributions and interactions of which require further investigation.

## Conclusion

**Figure 9:**
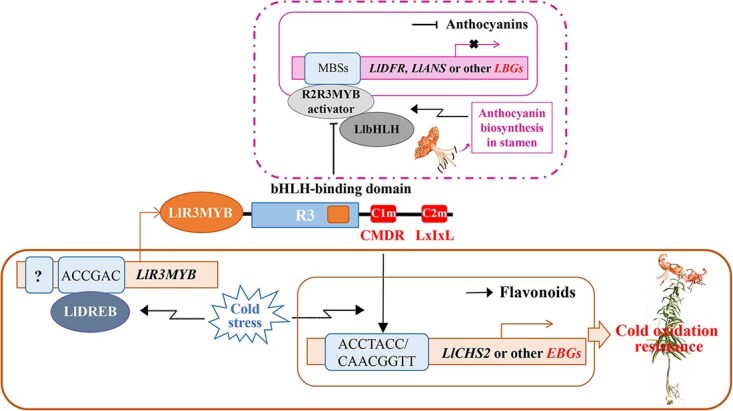
Model for the regulation of anthocyanin repressor LlR3MYB-mediated flavonoid accumulation in response to cold stress. *LlR3MYB* shows preferential expression in anthocyanin-pigmented floral tissues under normal condition, where it competitively inhibits the R2R3-MYB/bHLH activator complex formation, leading to downregulation of late biosynthesis genes (e.g. *LlDFR* and *LlANS*) and consequent suppression of anthocyanin accumulation. Low temperature and LlDREB induce LlR3MYB to directly bind to the promoter of *LlCHS2* or other early biosynthesis genes, thereby activating its expression and resulting in an increase in flavonoid accumulation. The enhanced accumulation of flavonoids improves the cold oxidative resistance of tiger lily plants.

LlR3MYB represents the first identified R3-MYB repressor that positively regulates cold-induced flavonoid accumulation in tiger lily (Fig. 9). This functional variation may be attributed to specific mutations in the C-terminal repression motifs of LlR3MYB. As a flavonoid activator, *LlR3MYB* exhibits strong cold induction mediated by LlDREB. This regulatory cascade upregulates *LlCHS2* or other EBGs, promoting the accumulation of ROS-scavenging flavonoids that mitigate oxidative stress. Thus, LlR3MYB functions as a molecular nexus integrating cold stress signaling with flavonoid biosynthesis via the ‘LlDREB-LlR3MYB-LlCHS2’ regulatory module.

## Materials and methods

### Plant materials and growth conditions

Potted and in vitro plants of the tiger lily (*Lilium lancifolium*) were planted in an experimental greenhouse (unheated and natural photoperiod) and a tissue culture laboratory (22°C, 16-h photoperiod, 1000 lux), respectively, of Central South University of Forestry and Technology, Changsha, China. Tobacco (*Nicotiana tabacum* k326 and *Nicotiana benthamiana*) were planted in a growth chamber set to 25 ± 2°C with a 16-h photoperiod.

### Gene cloning and sequence analysis

The coding sequences (CDS) of *LlR3MYB* (462 bp) (GenBank accession number: PV761027.1) and *LlDREB* (915 bp) (GenBank accession number: PV761028.1), along with the promoter sequences of *LlR3MYB* (1374 bp) and *LlCHS2* (931 bp), were amplified from tiger lily cDNA and genomic DNA. The primer sequences used in this study are provided in Table S10. For phylogenetic reconstruction, we obtained reference MYB, CHS, and DREB protein sequences from published data [11, 12, 28, 54, 81]. Protein evolutionary relationships were determined using MEGA11 software to construct maximum likelihood trees. Promoter cis-acting elements were predicted through PlantCARE database analysis.

### Stable transformation of tobacco and tiger lily

The CDS of *LlR3MYB* were cloned into pBWA(V)HS vector and subsequently transformed into *Agrobacterium tumefaciens* strain EHA105. Transgenic tobacco (*N. tabacum* k326) plants were generated using the established leaf disc method [93]. Tiger lily plant genetic transformation was performed as followed: In vitro aseptic slicing bulb scales (2–3 mm) of tiger lily were used as the explants. Explants were immersed in modified MS medium containing 100 μmol/l acetosyringone (AS) (OD_600_ = 0.4) for 15 min. Infected explants were transferred to co-culture medium (modified MS + 1.0 mg/l 6-BA +1.0 mg/l NAA + 100 μmol/l AS) and incubated in darkness for 48 h. The harvested scales were cultured on the bud differentiation medium (MS + 2.5 mg/l 6-BA+0.8 mg/l NAA + 30 g/l sucrose+100 mg/l vitamin C) and the root induction medium (MS + 1.5 mg/l NAA + 30 g/l sucrose), both containing 22 mg/l hygromycin B and 400 mg/l cefalexin for selection. In vitro surviving bulbs were collected for RT-PCR identification and incubate in GUS reaction buffer (Coolaber, Beijing, China).

### Virus-induced gene silencing

The 175-bp and 314-bp fragment of *LIR3MYB* and *LlDREB* were cloned into the TRV2 vector, respectively. The recombinant vector (TRV2-*LlR3MYB/LlDREB*) and empty vector controls (TRV1/TRV2) were introduced into the *A. tumefaciens* GV3101 strain. Bacterial cultures containing the silencing constructs were harvested and resuspended in infection buffer (10 mM MgCl_2_, 10 mM MES, and 200 μM AS, pH 5.6). The mixtures of infection buffer (OD_600_ = 1.0) containing TRV1: TRV2 (1:1, v/v) and TRV1: TRV2-*LlR3MYB/LlDREB* (1:1, v/v) the back side of leaves on eight-week-old cutting seedlings and of tepals on fully bloomed flowers. The treated plants were kept in the dark for 24 h and then transferred to a growth chamber (25 ± 2°C with a 16-h photoperiod) for 2 weeks. *LlR3MYB* transcript levels in silenced seedlings were quantified by qRT-PCR.

### Transient overexpression

The CDS of *LlDREB* was inserted into the pBWA(V)HS vector and subsequently transformed into *A. tumefaciens* strain GV3101. The procedure for transient overexpression of *LlDREB* followed the same infiltration protocol described above for virus-induced gene silencing in tiger lily seedlings.

### Cold stress treatment and qRT-PCR analysis

To investigate gene expression levels in different tissues or under cold stress, flowering tiger lily plants were subjected to 4°C treatment for 0, 1, 3, 6, 12, and 24 h. WT and *LlR3MYB*-overexpressed transgenic tobacco and tiger lily plants were subjected to 4°C treatment for 2 h. Samples were collected for qRT-PCR analysis, RT-PCR identification, and functional assessment. qRT-PCR was performed using the Talent qPCR PreMix SYBR Green (Tiangen, Beijing, China) and the Roche Lightcycler® 96 Instrument. The gene tonoplast intrinsic protein 41 (*LlTIP41*) from tiger lily or *Actin* gene from k326 tobacco was used as an internal standard. Gene expression was analyzed using the 2^–ΔΔCt^ method.

### Quantification of flavonoid contents

The total flavonoid, anthocyanin, and proanthocyanidin contents of fresh tissues of tiger lily or tobacco were detected using commercial assay kits following the manufacturer’s protocols (AKPL015C for flavonoid, AKPL021C for anthocyanin, and AKPL017C for proanthocyanidin; Boxbio Science, Beijing, China).

### Analysis of low-temperature oxidation resistance

One-month-old *LlR3MYB*-overexpressed tobacco and *LlR3MYB*-silenced tiger lily seedlings were treated 4°C for 2 h. Leaf samples were collected for total antioxidant capacity (T-AOC), superoxide dismutase (SOD), and catalase (CAT) activities, malondialdehyde (MDA) content and relative electrolyte conductivity (REC), along with histochemical staining using DAB and NBT. The T-AOC, SOD, and CAT activities, and MDA content were detected using commercial assay kits according to the manufacturers’ protocols (AKAO012C for T-AOC, AKAO001C for SOD, and AKAO003-1 U for CAT, AKFA013C for MDA; Boxbio Science, Beijing, China). The relative conductivity of fresh leaves (100 mg) was assessed in solution utilizing a conductivity detector for the evaluation of the REC.

### RNA isolation, sequencing, and transcriptome analysis

Total RNA extracted from in vitro-cultured tiger lily bulbs was used for cDNA library construction and RNA sequencing, as described before [94]. Transcript sequences after de-redundancy were aligned with KEGG databases using DIAMOND software. DEGs were identified by DESeq2, setting thresholds at |log_2_fold change (FC)| ≥ 1 and false discovery rate (FDR) < 0.05. PCA was computed using the prcomp in the R and visualized in OriginPro 2022.

### Protein isolation, characterization, and proteomic profiling

Protein samples were isolated from in vitro-cultured tiger lily bulbs, and their concentrations were analyzed using a BCA protein assay kit (Beyotime, China). Following established methods [95], the extracted proteins were enzymatically digested into peptides, which were subsequently separated and analyzed via NanoElute UHPLC system (Bruker Daltonics, Germany) coupled with timsTOF Pro 2 mass spectrometer (Bruker Daltonics, Germany). The mass spectrometry (MS) raw data were processed using DIA-NN software (v1.8.1) in library-free mode, followed by functional annotation against the KEGG database. The MaxLFQ algorithm was employed for protein quantification, with differential expression thresholds set at FC ≥ 1.5 or ≤ 0.6667 and *P* ≤ 0.05. KEGG enrichment was conducted using the clusterProfiler package in R, identifying significantly altered biological pathways based on the DEPs.

### Metabolite profiling and metabolic pathway analysis

Metabolites Metabolite profiling of in vitro-cultured tiger lily bulbs was performed by UPLC-ESI-MS/MS (ExionLC™ AD). Metabolite separation and detection were carried out following established methods [94, 96]. The acquired MS data were processed to determine precise retention times, accurate mass-to-charge ratios (*m*/*z*), and fragmentation patterns. Metabolite identification was achieved through both the proprietary MWDB database (Metware Biotechnology, Wuhan, China) and publicly available metabolite repositories. Differentially accumulated metabolites (DAMs) were selected based on Variable Importance in Projection (VIP) scores >1 (derived from OPLS-DA models) and |Log_2_FC| ≥ 1.0. R package MetaboAnalystR was used for OPLS-DA model generation and validation. Metabolite annotation was performed using the KEGG Compound database, followed by metabolic pathway analysis through KEGG Pathway database.

### Yeast-two-hybrid assay

The tiger lily leaf and tepal (after 2 h 4°C treatment) cDNA library in yeast was constructed by OE Biotech Co., Ltd. (Shanghai, China). The ORFs of LlR3MYB were cloned into the pGBKT7 bait vector. The bait and prey plasmids were separately introduced into Y2HGold and Y187 strains, respectively, via the Yeastmaker™ Yeast Transformation (Clontech, USA). Mated transformants were first cultured on SD/-Trp medium, then interactions were selected on selection plates (SD/-Leu/-Trp/-His/X-*α*-gal and -Ade variants). Transactivation was evaluated through growth capability and *α*-galactosidase activity, as described in the Matchmaker™ Gold Yeast Two-Hybrid System (Clontech, USA) user manual.

### Bimolecular fluorescence complementation (BiFC) assay

The coding sequences of full-length LlR3MYB and LlbHLH were inserted into the pSPYNE173 and pSPYCE(M) vectors, respectively. The resulting constructs, along with empty vector controls, were introduced into *A. tumefaciens* strain GV3101 for transient expression via *N. benthamiana* leaf infiltration. Plants were then initially kept in darkness for 24 h and subsequently transferred to a 16 h light/8 h dark photoperiod for an additional 32 h. Images were generated through Leica TCS SP8 Confocal Laser Scanning Platform using a 500–530 nm emission filter.

### Yeast-one-hybrid assay

The Y1HGold yeast strain was co-transformed with the pAbAi bait vector containing the *LlR3MYB* promoter region, and the pGADT7-Rec prey vector carrying tiger lily cDNA library ORFs. Transformants were selected on SD/-Ura plates with 500 ng/ml aureobasidin A (AbA; Sigma-Aldrich). Positive clones were sequenced and subsequently validated through pairwise Y1H assays using the *LlR3MYB* promoter-containing pGADT7-Rec prey vector.

### Dual-luciferase reporter assay

To evaluate the transcriptional activity of LlR3MYB and LlDREB, the Dual-Luciferase Reporter (DLR) Assay System (Promega, USA) was employed in tobacco protoplasts and leaves. Firefly (LUC) and Renilla (REN) luciferase activities were measured, and promoter activity was quantified as the LUC/REN ratio. The CDS of *LlR3MYB*, along with a series of its mutant variants, was fused to the GAL4 DNA-binding domain (BD) under the control of the 35S promoter. The GAL4-BD fusion was used as a negative control. Both the LlR3MYB-BD and GAL4-BD effector constructs were transiently expressed in tobacco protoplasts [97]. Additionally, the open reading frames (ORFs) of *LlDREB* and *LlR3MYB*, together with the series of *LlR3MYB* mutant variants, were cloned into the pGreenII 62-SK effector vector. The promoters of *LlR3MYB* and *LlCHS2* were individually inserted into the pGreenII 0800-LUC reporter vector. The constructed vectors and empty vector controls were transformed into *A. tumefaciens* strain GV3101 for *N. benthamiana* leaf infiltration. After 72 h, half of the infiltrated leaves underwent cold treatment (4°C, 24 h) before fluorescence signal detecting.

### EMSA analysis

EMSAs were performed following Li *et al*. [98]. The *LlR3MYB* and *LlDREB* ORF was cloned into pET-SUMO for MBP-tagged protein expression in *Escherichia coli* BL21(DE3). Purified MBP-LlR3MYB and MBP-LlDREB protein was incubated with biotin-labeled *LlCHS2* and *LlR3MYB* promoter probes containing the AC-I (ACCTACC), MBSI (CAACGGTT), and DRE (ACCGAC) cis-elements (Table S10), along with corresponding mutated controls. All steps followed the LightShift™ Chemiluminescent EMSA Kit protocol (Thermo Scientific, Shanghai, China).

## Supplementary Material

Web_Material_uhag065

## Data Availability

All data can be found within the manuscript and supporting materials.
