# Supplementary material for: LlR3MYB-mediated flavonoid biosynthesis confers cold stress tolerance in Lilium lancifolium through the LlDREB-LlCHS2 regulatory cascade
Source: Hortic Res. 2026 Feb 27;13(6):uhag065. doi: 10.1093/hr/uhag065 (PMC13249511; doi:10.1093/hr/uhag065)
Supplement: Web_Material_uhag065 [file web_material_uhag065.zip › Supplementary Figure.docx]

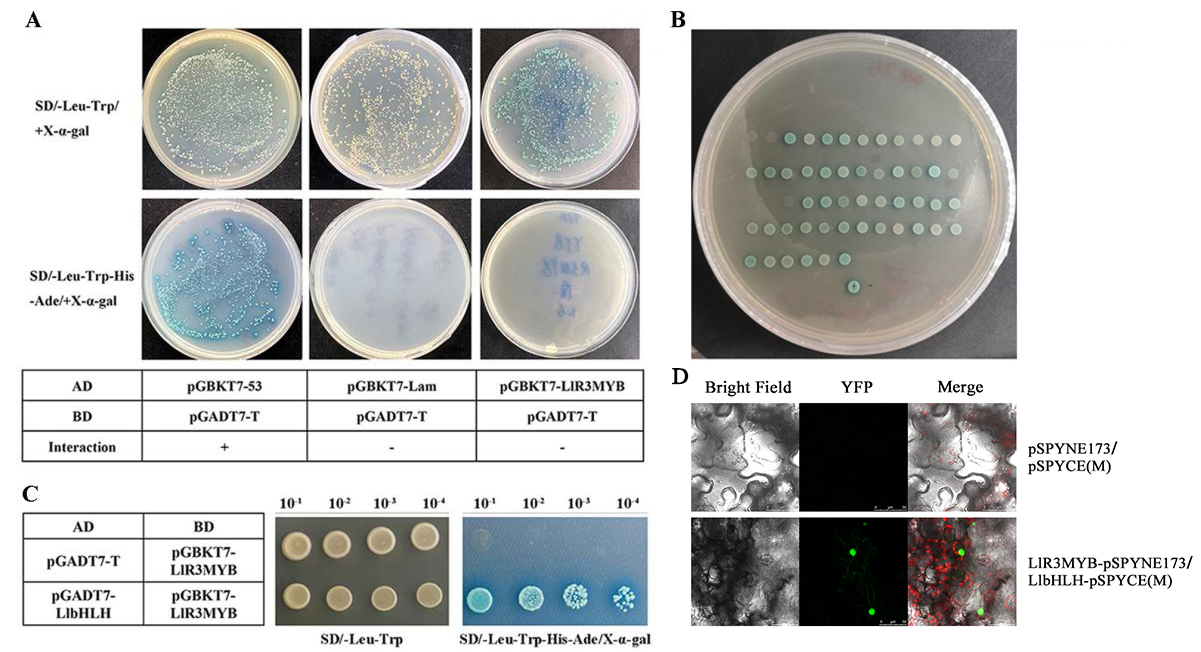


**Figure S1. Yeast-two-hybrid and BiFC assay of the interaction of LlR3MYB with LlbHLH.**

1. Self-activation and toxicity detection of pGBKT7-LlR3MYB.
2. Positive clones on rescreening (SD/-Trp-Leu-Ade-His/-X-α-Gal/-AbA medium) plate. Information of selected candidate proteins from yeast two-hybrid screening for interactions with LlR3MYB was shown in Table S1.
3. Verification of the interaction between LlR3MYB and LlbHLH. The yeast cells expressing the indicated proteins were plated onto selective media: SD/-Leu-Trp, SD/-Leu-Trp/-X-α-gal, and SD/-Leu-Trp-His-Ade/-X-α-gal. Co-transformation of pGADT7-53/pGBKT7 and pGADT7-Lam/pGBKT7 empty vectors (AD/BD) were used as positive and negative control, respectively.
4. BiFC assay using tobacco epidermal cells. Negative control was pSPYNE173/pSPYCE (M).


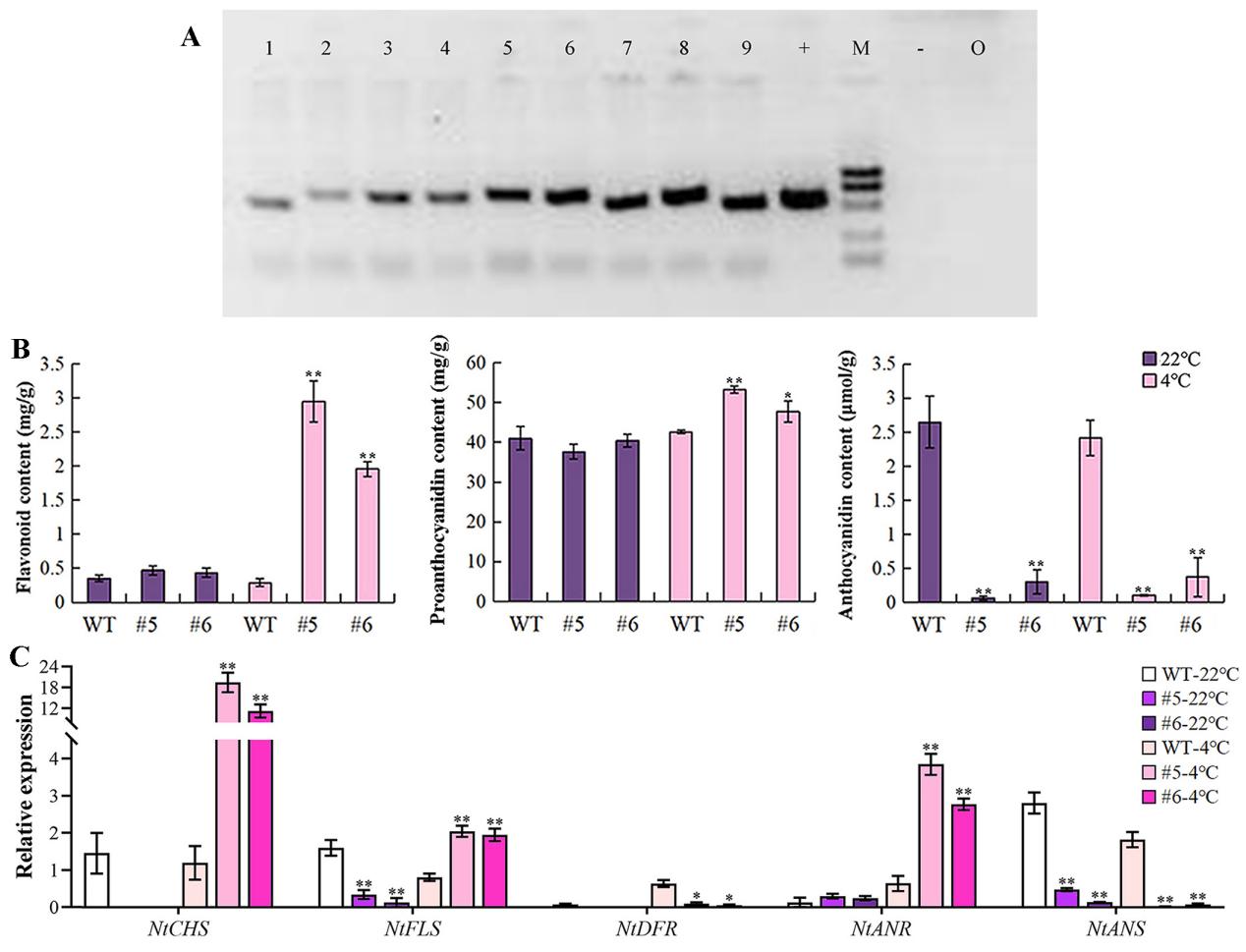


**Figure S2. Phenotypic and molecular characterization of cold stress responses in *LlR3MYB*-overexpressing transgenic tobacco plants.**

1. PCR detection of *LlR3MYB* transgenic tobacco. (M) Marker; (+) positive control; (-) negative control; (O) empty control; (1-9) transgenic tobacco lines. Line 5 and 6 were chosen for subsequent experiments.
2. Quantification of total flavonoids, proanthocyanidins, anthocyanins in WT and transgenic tobacco flowers under control (22°C) and cold (4°C) conditions.
3. qRT-PCR analysis of flavonoid pathway gene expression in WT and transgenic tobacco flowers at 22°C and 4°C. Values are mean ± SD from three independent biological replicates (^*^ *P* < 0.05; ^**^ *P* < 0.01; t-test).


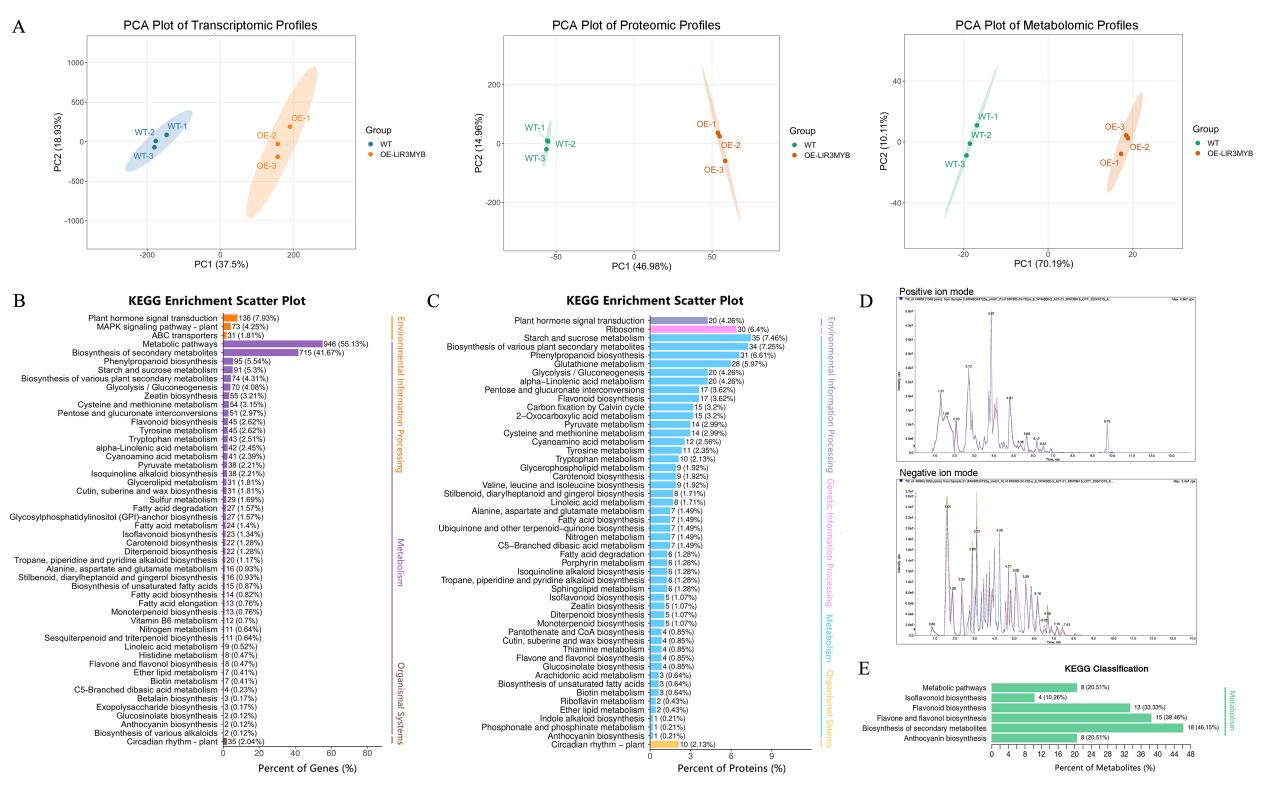


**Figure S3. Transcriptome, proteome, and metabolome analysis of LlR3MYB overexpressing tiger lily.**

1. Principal component analysis (PCA) of the transcriptomic, proteomic, and metabolomic profiles of *LlR3MYB*-overexpressing tiger lily (OE-LlR3MYB) and wild type (WT) samples.

(B, C) KEGG pathway enrichment analysis of the (B) differentially expressed genes (DEGs) and (C) differentially expressed proteins (DEPs).

1. Total ions current of one quality control sample by mass spectrometry.
2. KEGG classification of the differentially accumulated metabolites (DAMs).

**
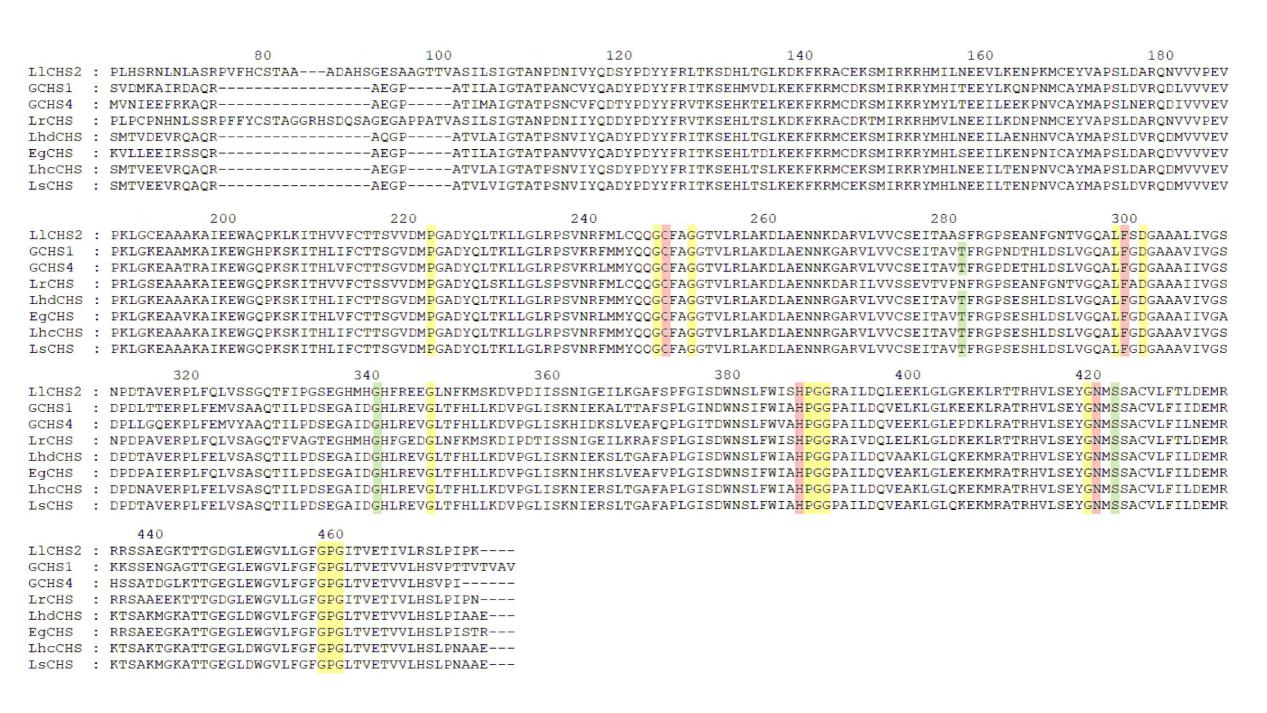
**

**Figure S4. The amino acid sequence alignment for the LlCHS2**.

Alignment with known CHS sequences (*Gerbera hybrida* GCHS1/4 (Deng *et al*., 2014), *Lilium regale* LrCHS (ASV46326.1), *L.* hybrid division I LhCHS1 (AZL57041.1) and LhCHS (BAB40786.2), *Elaeis guineensis* EgCHS, XP 010929491.1, and *L. speciosum* LsCHS, BAE79201.1) demonstrated that LlCHS2 preserved the conserved catalytic residue (Cys244, Phe295, His383, Asn416; marked in red), all 13 inert active site residues (marked in yellow), and the three critical binding pocket residues (Thr277, Gly336, Ser418; marked in green) that define the 4-coumaroyl-CoA and polyketide cyclization sites, with Thr277 substituted by Ser in LlCHS2 and Asn in LhdCHS.


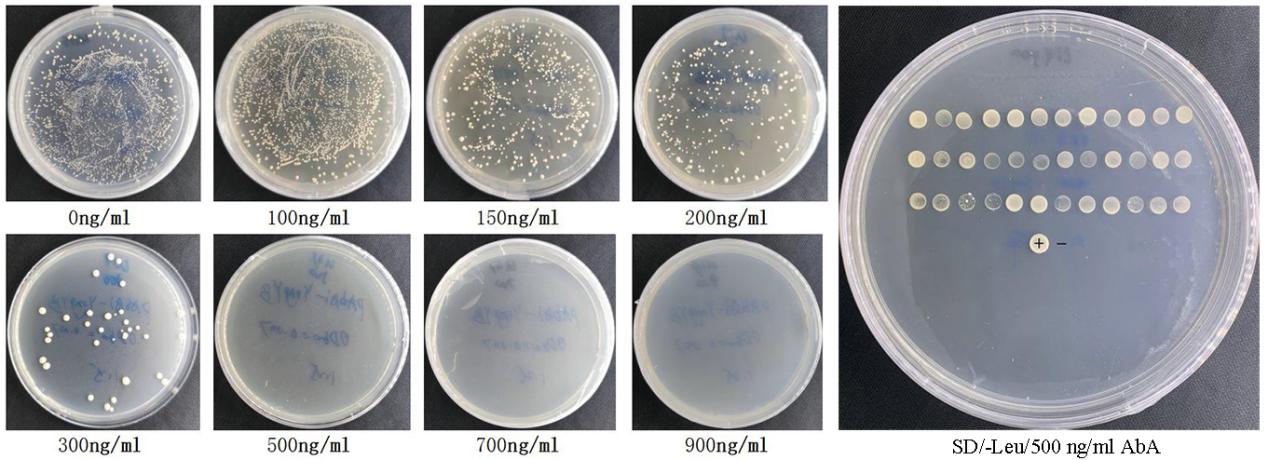


**Figure S5. Aureobasidin A (AbA) concentration selection and secondary screening for pAbAi-*LIR3MYBpro*.**

The minimal inhibitory concentration of AbA for bait yeast strains was found to be 500 ng/mL and 22 clones were observed being survived on selective medium.

**
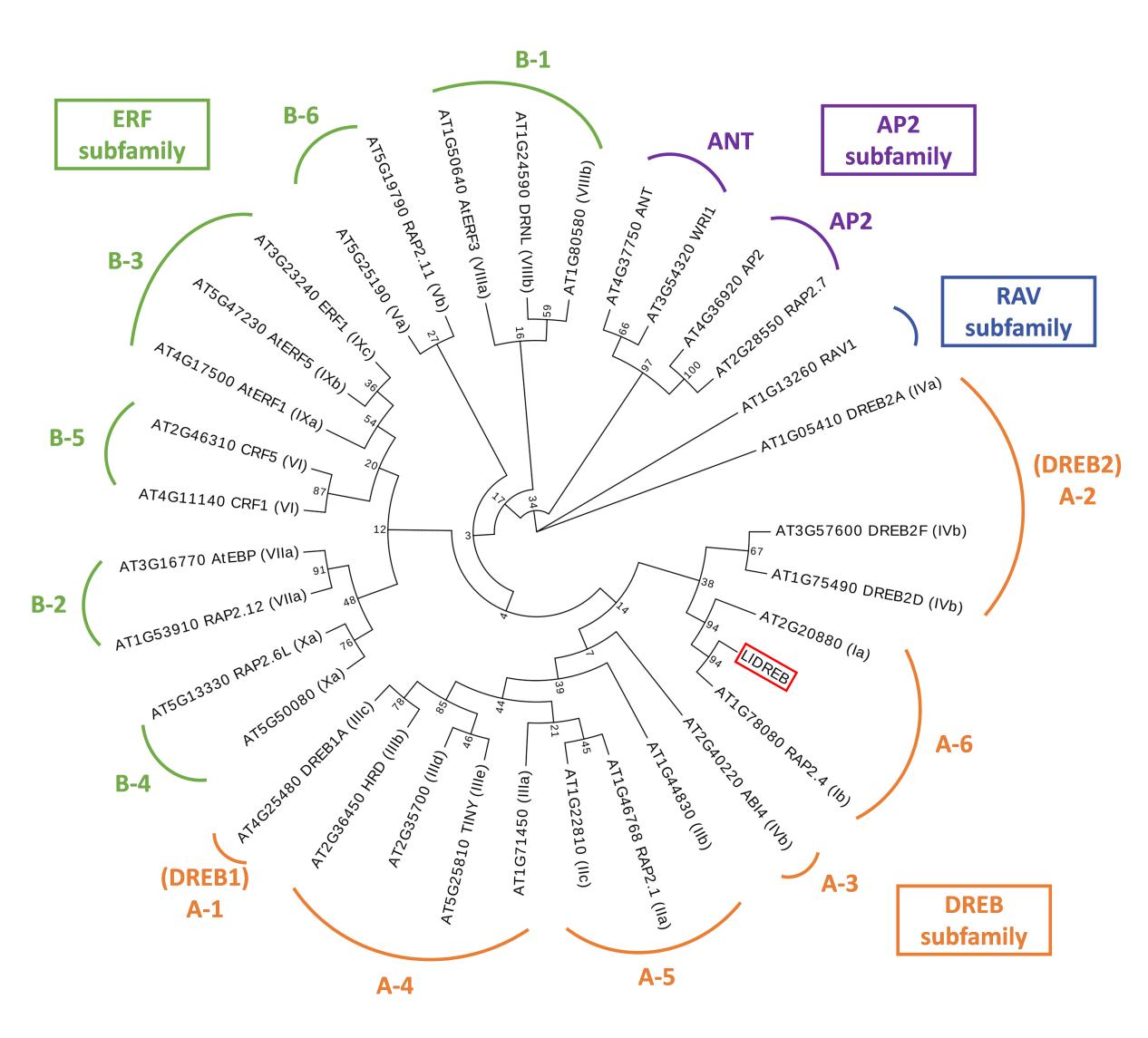
**

**Figure S6. Phylogenetic tree of LlDREB and the AP2/ERF family transcription factors in *Arabidopsis thaliana***.

LlDREB are marked by the red box. The deduced amino acid sequences were retrieved from the Tair database.

**
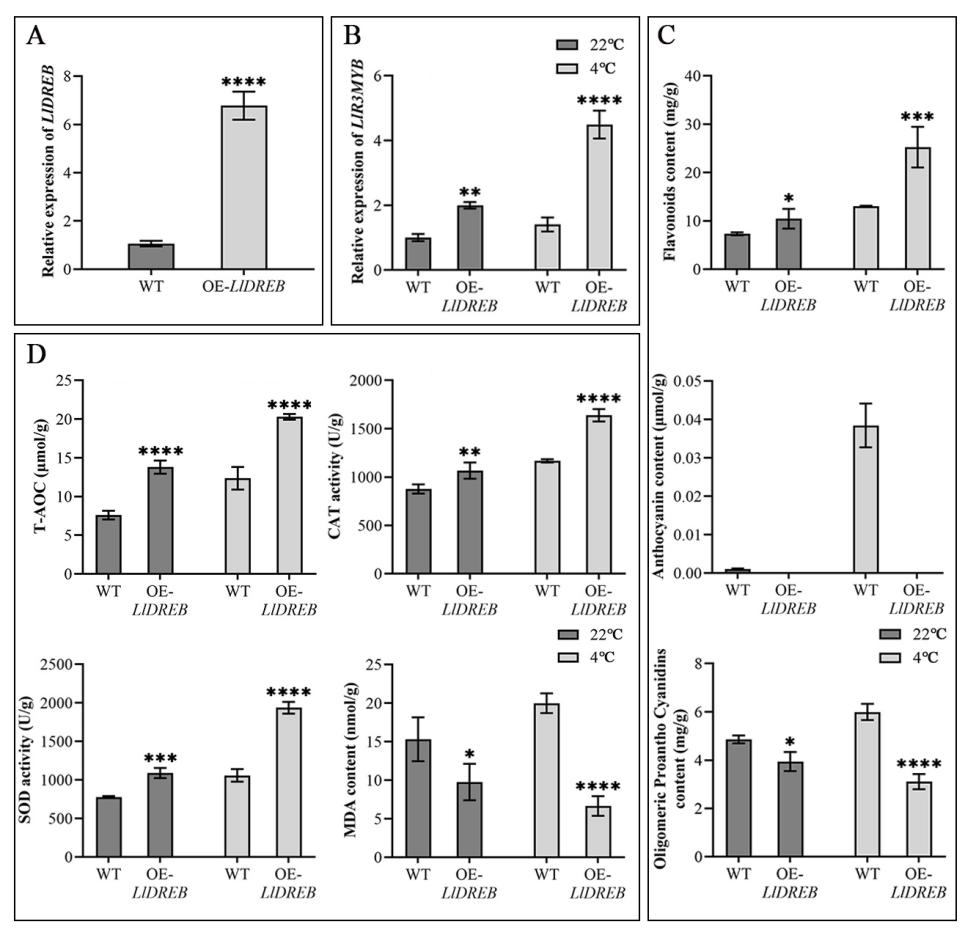
**

**Figure S7. Cold stress response phenotypes of *LlDREB*-overexpressed tiger lily cutting seedlings.**

1. Validation of *LlDREB* overexpressing by qRT-PCR.
2. qRT-PCR analysis of *LlR3MYB* gene expression.
3. Quantification of total flavonoids, proanthocyanidins, and anthocyanins in WT and transgenic plants under control (22°C) and cold (4°C) conditions.

(D) Biochemical analyses of antioxidant capacity (T-AOC, SOD, CAT activity), and MDA levels in WT and transgenic plants under control (22°C) and cold (4°C) conditions.

Values are mean ± SD from three independent biological replicates (^*^ *P* < 0.05; ^**^ *P* < 0.01; ^***^ *P* < 0.001; ^****^ *P* < 0.0001; t-test).
